# Recurrent Acute Pancreatitis in the Setting of Abnormal Pancreaticobiliary Junction

**DOI:** 10.7759/cureus.47029

**Published:** 2023-10-14

**Authors:** Kais Antonios, Neil Shah, Timothy McGorisk

**Affiliations:** 1 Internal Medicine, Trinity Health Ann Arbor, Ann Arbor, USA; 2 Gastroenterology, Trinity Health Ann Arbor, Ann Arbor, USA

**Keywords:** rap, anomalous union of pancreaticobiliary junction, abnormal pancreaticobiliary junction, recurrent acute pancreatitis, apbj

## Abstract

Anomalous or abnormal pancreaticobiliary junction (APBJ) is an important structural cause of recurrent acute pancreatitis. Outside of the common causes of recurrent acute pancreatitis, such as alcohol, gallstones, or hypertriglyceridemia, this anatomical variant can often be overlooked and lead to delays in patient care and even mismanagement. It can be defined as the abnormal junction of the pancreatic duct and common bile duct that occurs outside the duodenal wall to form a long common channel (>8 mm). We describe a case of a 51-year-old female with multiple episodes of acute pancreatitis. Further investigation led to the diagnosis of an aberrant pancreatic duct anatomy with the common bile duct measuring around 20 mm. This report will include a discussion about the pancreaticobiliary junction, how it can be diagnosed, and what complications it can precipitate.

## Introduction

Abnormal pancreaticobiliary junction (APBJ) is a rare condition that can pose a diagnostic challenge. Certain types can cause recurrent acute pancreatitis (RAP). Normally, the major pancreatic duct and the common bile duct open into the second part of the duodenum alone or after joining as a common channel [1]. The junction of the common bile duct and pancreatic duct is crucial for sphincter control of bile and pancreatic juice drainage. However, bidirectional regurgitation can occur if the union is above the sphincter of Oddi. An APBJ happens when the common bile duct and the main pancreatic duct join outside the wall of the duodenum and form a long common channel (>8 mm) [2,3]. In this case, the common channel was approximately 20 mm long. This aberrant anatomy is the likely culprit in this patient’s recurring pathology after an in-depth investigation ruled out more common etiologies of RAP.

Prior presentations: This article was presented as a poster presentation at the American College of Gastroenterology meeting in Charlotte in 2022.

## Case presentation

A 51-year-old female with a past medical history of hypertension, hyperlipidemia, chronic kidney disease, obstructive sleep apnea, and recurrent episodes of acute pancreatitis. She presented with typical epigastric pain radiating to her back. Laboratory workup included revealed an elevated lipase level of 5131 U/L (Reference range 11-82 U/L). Upon review of her medical history, this was the sixth documented episode of acute pancreatitis in the past six years. All her prior episodes presented similarly with typical epigastric pain radiating to her back, with elevated lipase to >3 times the upper limit of normal. The most recent presentation was 9 months prior. The patient remained hemodynamically stable during her past hospitalizations and never required a transfer to an intensive care unit. She reliably denied any significant alcohol use nor did she have chronic alcohol use during any of these episodes. Multiple abdominal ultrasounds and cross-sectional images did not show signs of cholelithiasis or choledocholithiasis, additionally, a semi-elective cholecystectomy was being considered for possible microlithiasis. A magnetic resonance cholangiopancreatography (MRCP) was performed two years prior to the latest presentation, which ruled out any obstructive etiology in the biliary tract that could be attributable to the patient’s recurrent presentation. However, it showed that the pancreatic duct appeared to join the common bile duct along its mid-course, compatible with a long common channel bile duct measuring approximately 20 mm. Additionally, her imaging revealed mild pancreatic and peripancreatic edema which was suspicious for acute pancreatitis.

**Figure 1:**
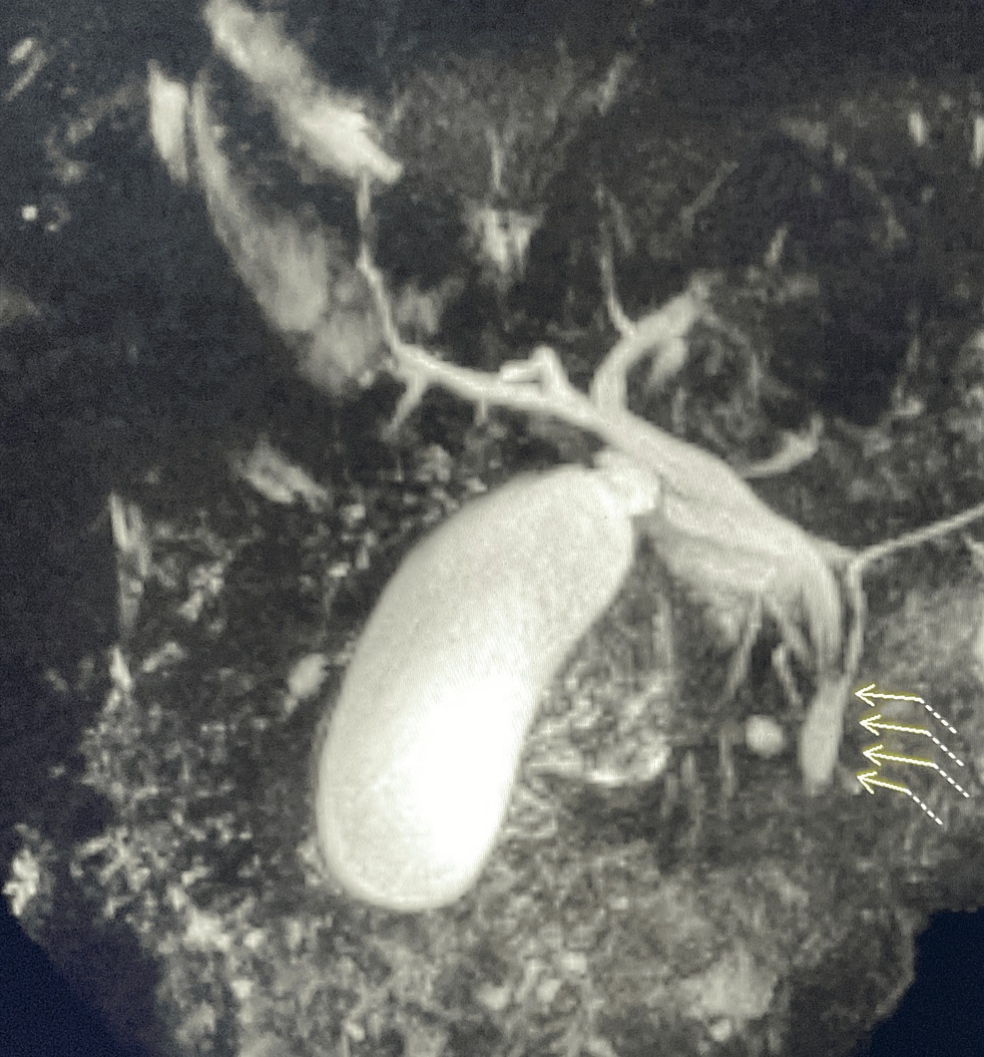
Aberrant pancreatic duct anatomy with a long common channel of the distal bile duct and the main pancreatic duct measuring approximately 24 mm in length (Yellow arrows). The common hepatic duct and the common bile duct are obscured by fluid in the duodenum but are nondilated.

**Figure 2:**
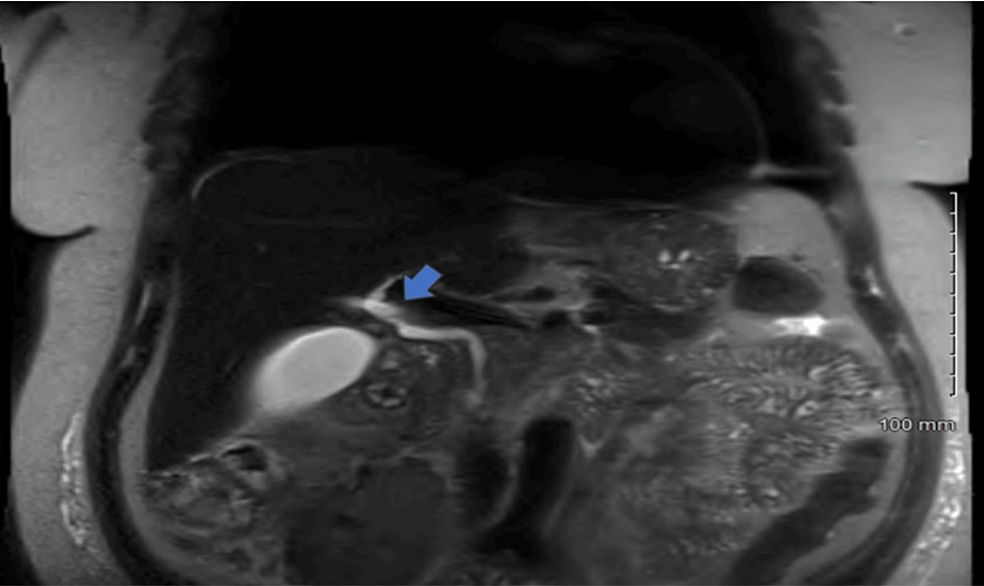
Higher resolution coronal view showing the aberrant pancreatic duct anatomy with a long common channel of the distal bile duct and the main pancreatic duct measuring approximately 24 mm in length (Blue arrow)

A repeat MRCP during this hospitalization demonstrated the finding of an aberrant pancreatic duct anatomy with a long common channel of the distal bile duct and the main pancreatic duct measuring approximately 24 mm in length (Figures 1-2), with the common bile duct measuring 8 mm in caliber at the porta hepatis. Surgical and endoscopic interventions including sphincterotomy were discussed initially, but given the patient’s rapid clinical improvement, she was recommended to follow up with hepatobiliary surgery as an outpatient. She continued to follow up with gastroenterology as an outpatient at which time genetic testing for hereditary pancreatitis was performed and her sequencing panel (CFTR, CTRC, PRSS1, SPINK1) was negative. The patient has not followed up with surgery since being discharged from the hospital, though she has remained free of acute pancreatitis episodes for more than a year at the time of writing this paper.

## Discussion

RAP is a clinical condition characterized by repeated episodes of acute pancreatitis; RAP is therefore diagnosed retrospectively by clinical definition after at least two episodes of acute pancreatitis [4]. There are several etiologies of RAP especially if the underlying cause is not definitively addressed.

APBJ is an important cause of RAP, along with alcohol use and gallstones [5]. One of the studies that looked at the prevalence of APBJ was conducted by Takuma et al., where the morphology of the pancreaticobiliary tract was examined in 230 of 381 patients with acute pancreatitis using endoscopic retrograde cholangiopancreatography. RAP was diagnosed in 74 patients (19%). APBJ was found to be the third most common cause of RAP after alcoholic pancreatitis and idiopathic etiology [5]. The reported frequency of ABPJ ranged from 1.5% to 3.2% in different ethnic populations [6]. RAP with APBJ had a male-to-female ratio of 1:4.3. Interestingly, the recurrence rate for acute pancreatitis was at its highest among patients with APBJ, significantly higher than any other cause for RAP [5].

The clinical features of APBJ vary between different patients. Whilst some might experience RAP, others can be asymptomatic. Certain features on radiographic imaging -whether MRCP or ERCP- have been associated with a higher incidence of acute pancreatitis, which include a long (>21 mm) and wide (>5 mm) common channel, a wide diameter of the proximal pancreatic duct (>2.5 mm), the presence of a filling defect in the common channel, and the presence of a pancreatic duct anomaly [7,8].

In addition to acute pancreatitis, APBJ junction has been associated with other pancreatic disorders including chronic calcific pancreatitis and pancreatic carcinoma [3,7]. Gallbladder carcinoma has also been observed with APBJ, as the reflux of pancreatic juice into the gallbladder may cause increased bile pressure, and result in epithelial hyperplasia [7,9].

Regardless of the etiology of acute pancreatitis. Initial management should focus on fluid resuscitation, with some data to support Ringer’s lactate over physiologic saline. Routine use of prophylactic antibiotics in acute pancreatitis is not recommended, nor is urgent endoscopic retrograde cholangiopancreatography in the absence of concomitant acute cholangitis. Early oral feeding should be encouraged, not avoided, and the use of parenteral nutrition is discouraged [10].

As medical management without surgical intervention would not reverse the underlying pathology and pathophysiology of the disease, most interventional options for APBJ have been surgical. With the associated increased risk for cholangiocarcinoma associated with APBJ, surgical options have included a prophylactic cholecystectomy even in the absence of evidence of an underlying malignancy [11]. A prophylactic cholecystectomy has shown benefit in certain studies whether a choledochal cyst is present or not [12,13]. Additionally, a hepaticojejunostomy could be performed depending on the presence or absence of an accompanying choledochal cyst. In a study conducted by Sugiyama et al., 64 adults with APBJ (43 with and 21 without choledochal cyst) underwent surgical treatment. All patients with choledochal cysts underwent cyst excision and hepaticojejunostomy. While patients without choledochal cysts or pancreaticobiliary carcinoma underwent cholecystectomy alone. No patients experienced pancreatitis during a mean postoperative follow-up of 6.7 years [7]. On the other hand, total resection of the extrahepatic bile duct and hepaticojejunostomy is recommended in children diagnosed with APBJ. Early diagnosis and early surgical treatment provide a good prognosis with few complications [14].

Another treatment approach to APBJ that has been discussed in the literature, though mostly in smaller scale studies, is endoscopic sphincterotomy (ES) in a selected population to possibly serve as a bridge to surgery, especially with patients with active obstructive jaundice, chronic pancreatitis, or RAP, as preoperative ES could improve drainage, resolve complications allow for a subsequent safe operation, and guarantees postoperative pancreatic drainage [15,16]. This was demonstrated in a study done in 2008 by Terui et al. where ES was performed in four pediatric patients with APBJ and refractory acute pancreatitis defined as pancreatitis that worsened in spite of appropriate medical treatments [16]. These patients had a significant improvement in their symptoms and later underwent a cholecystectomy followed by a choledochoduodenostomy. They all had a favorable postoperative course with no complications in 2-10 years follow-ups. In a more recent study in 2023, similar results were demonstrated in five patients with APBJ and either RAP or chronic pancreatitis, though the follow-up period was only 6 months [17].

## Conclusions

APBJ should be considered an important structural cause of RAP because if recognized early it can lead to implementing treatment strategies to reduce the burden of recurrence and complications on the patient and may even help reduce repeated admissions. MRCP is a noninvasive and accurate imaging method for diagnosis. Treatment options should consider surgical interventions including a hepaticojejunostomy and a prophylactic cholecystectomy. An evaluation for a preoperative ES might be helpful in patients with chronic pancreatitis, or RAP unresponsive to medical treatment.

## Appendices

This article was presented as a poster at the American College of Gastroenterology meeting in Charlotte 2022 [18].
